# Estimation of Nitrogen Nutrition Status in Winter Wheat From Unmanned Aerial Vehicle Based Multi-Angular Multispectral Imagery

**DOI:** 10.3389/fpls.2019.01601

**Published:** 2019-12-06

**Authors:** Ning Lu, Wenhui Wang, Qiaofeng Zhang, Dong Li, Xia Yao, Yongchao Tian, Yan Zhu, Weixing Cao, Fred Baret, Shouyang Liu, Tao Cheng

**Affiliations:** ^1^National Engineering and Technology Center for Information Agriculture (NETCIA), MARA Key Laboratory for Crop System Analysis and Decision Making, Jiangsu Key Laboratory for Information Agriculture, Jiangsu Collaborative Innovation Center for Modern Crop Production, Institute of Smart Agriculture, Nanjing Agricultural University, Nanjing, China; ^2^UMR EMMAH, INRA, UAPV, Avignon, France

**Keywords:** multi-angular, unmanned aerial vehicle, vegetation index, nitrogen status, zenith angle, wheat

## Abstract

Rapid, non-destructive and accurate detection of crop N status is beneficial for optimized fertilizer applications and grain quality prediction in the context of precision crop management. Previous research on the remote estimation of crop N nutrition status was mostly conducted with ground-based spectral data from nadir or oblique angles. Few studies investigated the performance of unmanned aerial vehicle (UAV) based multispectral imagery in regular nadir views for such a purpose, not to mention the feasibility of oblique or multi-angular images for improved estimation. This study employed a UAV-based five-band camera to acquire multispectral images at seven view zenith angles (VZAs) (0°, ± 20°, ± 40° and ±60°) for three critical growth stages of winter wheat. Four representative vegetation indices encompassing the Visible Atmospherically Resistant Index (VARI), Red edge Chlorophyll Index (CI_red-edge_), Green band Chlorophyll Index (CI_green_), Modified Normalized Difference Vegetation Index with a blue band (mND_blue_) were derived from the multi-angular images. They were used to estimate the N nutrition status in leaf nitrogen concentration (LNC), plant nitrogen concentration (PNC), leaf nitrogen accumulation (LNA), and plant nitrogen accumulation (PNA) of wheat canopies for a combination of treatments in N rate, variety and planting density. The results demonstrated that the highest accuracy for single-angle images was obtained with CI_green_ for LNC from a VZA of -60° (*R^2^* = 0.71, RMSE = 0.34%) and PNC from a VZA of -40° (*R^2^* = 0.36, RMSE = 0.29%). When combining an off-nadir image (-40°) and the 0° image, the accuracy of PNC estimation was substantially improved (CI_red-edge_: *R^2^* = 0.52, RMSE = 0.28%). However, the use of dual-angle images did not significantly increase the estimation accuracy for LNA and PNA compared to the use of single-angle images. Our findings suggest that it is important and practical to use oblique images from a UAV-based multispectral camera for better estimation of nitrogen concentration in wheat leaves or plants. The oblique images acquired from additional flights could be used alone or combined with the nadir-view images for improved crop N status monitoring.

## Introduction

Nitrogen (N) status is a critical nutrient indicator in crop growth for optimizing fertilization management. Reasonable N fertilizer application can not only improve N use efficiency and crop yield, but also reduce environmental pollution (Fitzgerald et al., 2010). Leaf N concentration (LNC) and plant N concentration (PNC) are important indicators for N fertilizer application at early growth stages (Hansen and Schjoerring, 2003; Li et al., 2018) and they are highly related to the final grain quality (Zheng et al., 2018b). As a product of N concentration and biomass, the two nitrogen accumulation parameters leaf N accumulation (LNA) and plant N accumulation (PNA) are used not only to diagnose crop N status, but also to evaluate crop production capability and predict grain quality (Zheng et al., 2018a). Accurate, real-time and rapid detection of crop N status is beneficial for agricultural management practices and also helps guide efficient fertilizer applications (Hansen and Schjoerring, 2003; Yao et al., 2010). The measurement methods of N status for N concentration (%) and accumulation (g/m^2^) can be grouped into two categories: direct measurements based on chemical analysis and indirect estimation based on remote sensing. Although the traditional chemical methods can produce accurate measurements, the time-consuming, labor-intensive and destructive process constrains their applications to a large amount of samples and field conditions (Curran, 1989). In contrast, remote sensing has been proved to be an efficient tool and widely applied in rapid and non-destructive estimation of crop N status (Tilling et al., 2007; Li et al., 2010; Lee and Lee, 2013; Quemada et al., 2014; Yao et al., 2014).

In the past two decades, a number of methods have been proposed to estimate crop N status with spectral data, the most common one of which is spectral vegetation indices (VIs). The VI-based methods are often established through fitting relationships between N status parameters and VIs. It has proved to be an efficient and accurate method for monitoring N status because of its simplicity and ease of operation (Xue et al., 2004; Yao et al., 2014; Zhou et al., 2018). Previous studies on crops were mostly concerned about searching for the best VI and determining the optimal bands derived from hyperspectral data for monitoring N status in wheat (Hansen and Schjoerring, 2003; Yao et al., 2014; Yao et al., 2015), and rice (Tian et al., 2013; Zhou et al., 2018). Most of the VIs were determined from the bands in the visible and near infrared (VNIR) region due to the strong dependence between N and chlorophyll for fresh leaves and the deep absorption valleys of chlorophyll in the VNIR region (Curran, 1989; Li et al., 2018). At leaf level, a recent study by Li et al. (2018) demonstrated that the area-based N (N content) could be better estimated from reflectance than mass-based N (N concentration). Although N content better represents the interaction of matter and light per unit surface area, N concentration is preferred by agronomists for growth diagnosis and is still sought to be estimated more accurately from spectral data (Lemaire et al., 2008; Xia et al., 2014). At canopy level, the VIs based on VNIR bands could also be used to quantify N accumulation because it is highly related to crop biomass (Zheng et al., 2018a). Moreover, previous studies proved that the estimation of foliar biochemistry was affected by the crop canopy structure and sun-sensor geometry (Jay et al., 2017b). However, most studies still used canopy reflectance data from nadir observations, so that only the spectral information from the top layer of canopy could be considered. Some studies have demonstrated that the LNC of wheat decreased from top to bottom and the vertical N gradient for leaves was greater than that for stems (Dreccer et al., 2000; Wang et al., 2005). This vertical N distribution significantly affects the spectral differences among upper, middle, and lower layers of the canopy (Huang et al., 2011; Ye et al., 2018). With nadir observations, the estimation accuracy for crop N concentration remained relatively low compared to that for N accumulation (Jay et al., 2017b; Zheng et al., 2018b). Oblique or multi-angular observations might help detect N status more accurately due to its great potential in obtaining more information about the lower to upper layers of canopy than nadir observations, especially for open canopies (Pocewicz et al., 2007; Huang et al., 2011).

The attention to multi-angular remote sensing originated from its performance in improving the accuracy of land cover classification (Colstoun and Walthall, 2006; Koukal and Atzberger, 2012; Koukal et al., 2014). There are also many attempts to improve the estimation of crop growth or nutrition parameters using multi-angular remote sensing based on ground, airborne and spaceborne platforms. Generally, ground-based multi-angular observations were obtained with a goniometer system (Sandmeier and Itten, 1999; Sandmeier, 2000) or by manual operation of spectrometers at various view angles (He et al., 2016b; Song et al., 2016). With ground-based multi-angular measurements, it was found that the remotely sensed data from backward view (with the sensor facing away from the sun in the solar principal plane and often expressed in negative numbers) angles performed better than those from the nadir and forward view (with the sensor facing towards the sun in the solar principal plane and often expressed in positive numbers) angles in the estimation of crop parameters such as leaf nitrogen concentration (He et al., 2016b; Jay et al., 2017b). These studies indicated the potential of off-nadir observations in improving the estimation of crop nutrition parameters over conventional nadir observations. The ground systems could provide accurate angular sampling of canopy reflectance (Huang et al., 2011; He et al., 2016b; Song et al., 2016), but their inflexibility and low efficiency limit the applications to large areas in a timely manner. In contrast, multi-angular remote sensing from spaceborne platforms has been applied to large-scale mapping of crop and forest parameters (Schlerf and Atzberger, 2012). However, those spaceborne platforms might not be suitable for crop monitoring over small fields due to the deficiencies in spatial resolution, revisit frequency and data availability (Zheng et al., 2018a). The manned airborne platforms can collect data at higher temporal and spatial resolutions, but their operational complexity and cost hinder the frequent acquisition of multi-angular data for crop monitoring. With the advent of unmanned aerial vehicles (UAVs), aerial remote sensing systems become increasingly available for crop monitoring because of their flexibility, low cost, and ease of operation (Hunt et al., 2010; Roth and Streit, 2017; Zheng et al., 2018a; Lu et al., 2019).

To date, UAV-based multi-angular observations can be obtained in two ways. One is from nadir-viewing and highly overlapping images acquired from a frame camera (Roosjen et al., 2017). With such a system, multiple views of the same target on the ground could be extracted from the overlapping images based on geometry of camera positions on the flight track and pixel locations on the images. Koukal et al. (2014) has found that the multi-angular views obtained with this approach could improve forest classification compared to nadir observations alone. However, Roosjen et al. (2018) reported that the view angles derived from the overlapping images were not big enough and could not lead to significant improvement in the estimation accuracy of potato leaf area index (LAI) and leaf chlorophyll content (LCC). In addition, their simulations with the PROSAIL model indicated a substantial improvement when using spectra with view zenith angles up to 30° compared to those with nadir views alone. This means it is still possible to improve the estimation of crop parameters with UAV imagery as long as the images with large view angles are available. The other way is to acquire oblique UAV imagery by setting the camera viewing at various angles. Unlike the use of multiple oblique-viewing sensors on spaceborne platforms, a practical UAV platform usually carries only one lightweight camera due to its payload limitation and collects multi-angular imagery by adjusting the angle of gimbal upon request. However, such a multi-angular UAV system has rarely been used for crop monitoring and whether the estimation of crop nutrition parameters could be improved over nadir observations remains unclear. Examining the advantages of multi-angular or oblique images from UAV platforms has great potential in developing efficient and reliable solutions for monitoring crop N status in the context of precision farming.

Thus, the objectives of this study were 1) to assess the sensitivity of VIs derived from UAV imagery to crop N status of wheat at different view zenith angles (VZAs), and 2) to determine the optimal VZA and the VZA combination for the estimation of N nutrition parameters from UAV-based multi-angular imagery. Specially, we evaluated the performance of four representative VIs at seven VZAs for the estimation of LNC, PNC, LNA and PNA in wheat.

## Materials and Methods

### Experimental Design

The experiment was conducted at the experimental station of the National Engineering and Technology Center for Information Agriculture (NETCIA) located in Rugao, Jiangsu province of eastern China (120°45’ E, 32°16’ N) within the winter wheat season of 2016 to 2017. Two winter wheat cultivars with planophile and erectophile leaf types, *Yangmai 15* and *Yangmai 16* were seeded on November 15, 2016. Three N rates (0, 150, 300 kg/ha) with two planting densities (1.6 × 10^6^ plants/ha and 1.0 × 10^6^ plants/ha, corresponding to 0.25 m and 0.4 m row spacings) were applied with three replications. 50% of N fertilizers were applied at the sowing day and 50% at the jointing stage. A total of 36 plots with the size of 6 × 5 m^2^ each were used for the experiment and the plots were arranged in a randomized block design. Each plot was divided into ground sampling region and image analysis region. In order to avoid the complexity of soil N levels, the N level corresponded to the treatments of the preceding rice growing season for each plot.

### Data Acquisition

#### UAV-Based Multi-Angular Imagery

**Figure 1:**
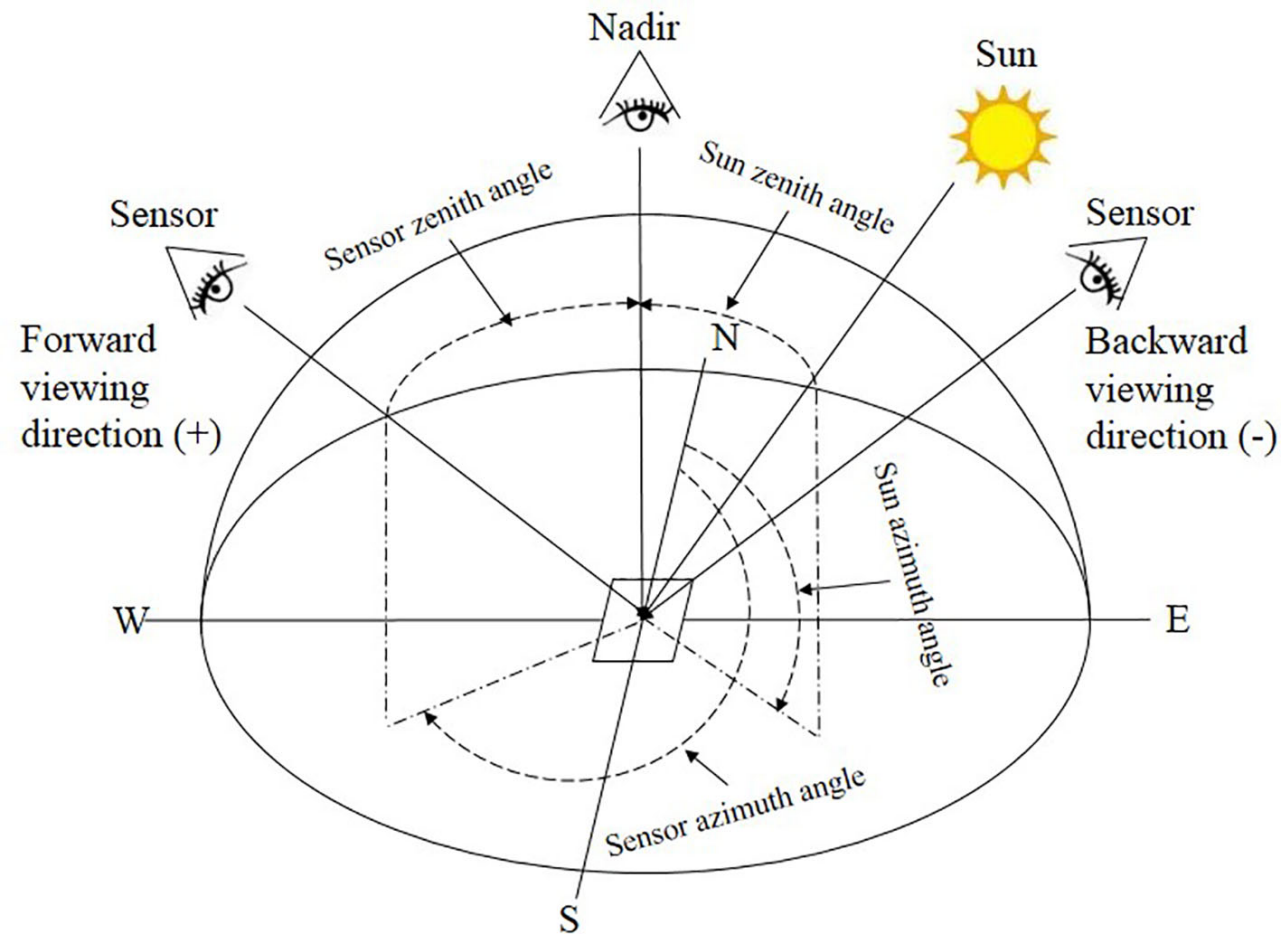
Schematic diagram of multi-angle remote sensing data acquisition.

**Figure 2:**
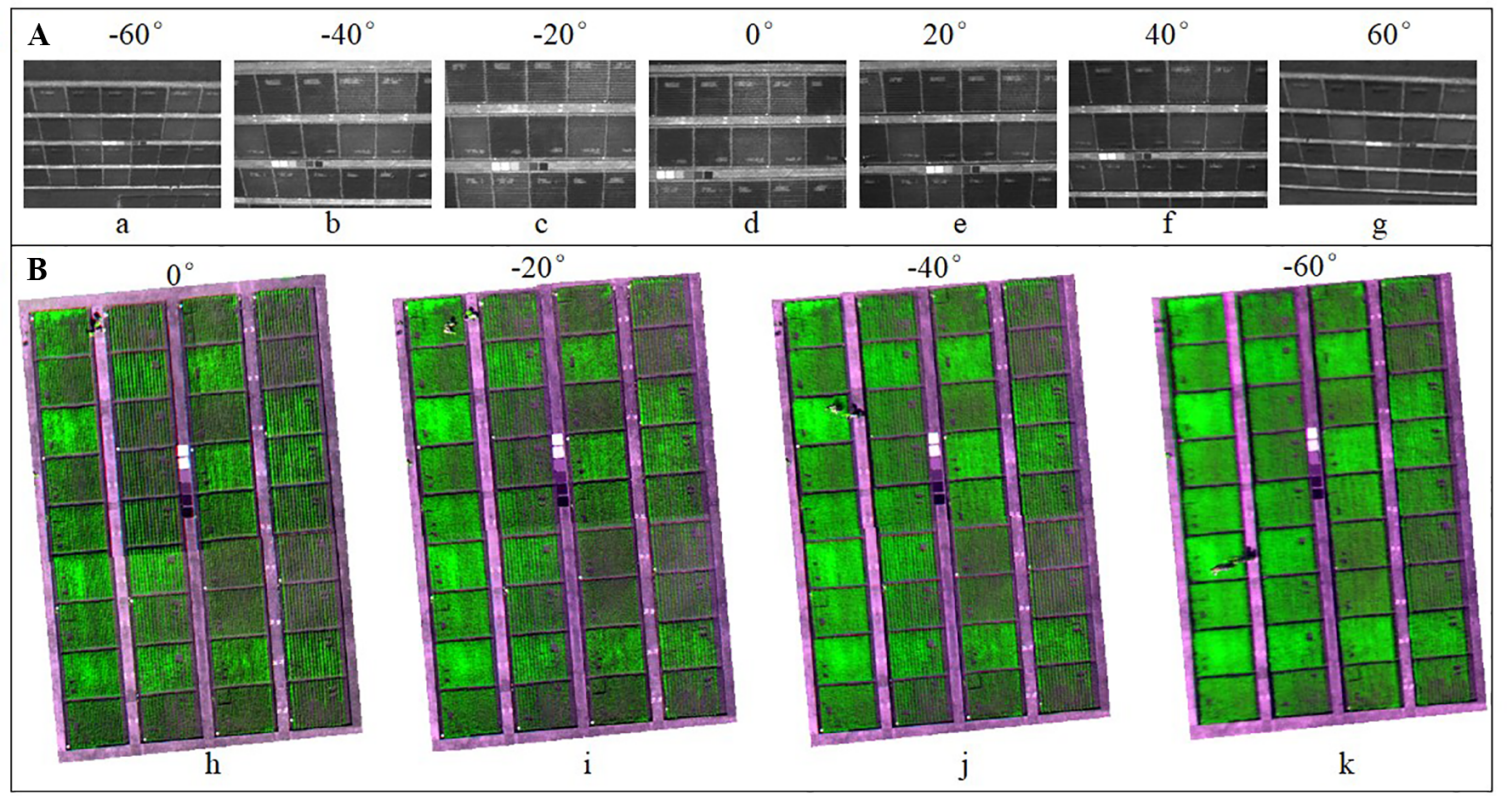
Images captured from a UAV-based multi-angular multispectral camera at the jointing stage. **(A)** represents the original images at seven angles (backward: a = -60°, b = -40°, c = -20°, Nadir: d = 0°, forward: e = 20°, f = 40°, g = 60°) at 800 nm captured with the UAV system. **(B)** represents the images at nadir observation (h) and backward view (i = -20°, j = -40°, k = -60°) in false color composition (700 nm, 800 nm, 671 nm).

This study employed a UAV system consisting of an eight-rotor Mikrokopter OktoXL UAV and a six-channel multispectral camera (Mini-MCA6, Tetracam, Inc., Chatsworth, CA, USA) to acquire multi-angular images (Yao et al., 2017; Zhou et al., 2017). The UAV had a maximum payload capacity of 2.5 kg and its flight duration was 8–25 min depending on the battery and actual payload. The Mini-MCA6 multispectral camera mounted onboard the UAV had an incident light sensor and five spectral bands with center wavelengths at blue (490 nm), green (550 nm), red (671 nm), red edge (700 nm), and near-infrared (800 nm). The incident light sensor channel was designed for calibration purposes and not for collecting aerial images. The field of view (FOV) of the Mini-MCA6 is 38.26° in the horizontal view and 30.97° in the vertical direction. The image size captured by this camera is 1280 × 1024 pixels. The camera was set to a 1.5 s shutter release interval to capture images with a 10-bit RAW format. In order to acquire multi-angular images, we fixed the looking angles of the camera with a goniometer before each flight. As shown in **Figure 1**, the view zenith angle (VZA) of the UAV camera was defined as 0° at nadir observation. While the VZA with the sensor facing towards the sun in the solar principal plane was defined as the forward direction and expressed in positive numbers, the VZA with the sensor facing away from the sun was defined as the backward direction and expressed in negative numbers. Seven VZAs, encompassing three in the backward (-60°, -40°, -20°), nadir (0°) and three in the forward (20°, 40°, 60°) viewing directions were sampled in this experiment (**Figure 2**). The wheat seeds were sowed along North-South direction. In order to reduce the effects of sun zenith angle (SZA) and sun azimuth angle (SAA) variations on reflectance, we used symmetric solar positions between morning and afternoon to acquire the backward-viewing and the forward-viewing images. To georeference the 5-band images from the multispectral camera for each growth stage, 25 ground control points (GCPs) were marked evenly on the concrete roads across the study site and its geographic coordinates were obtained from RTK-GPS (Real-Time Kinematic Global Positioning System, CHC X900 GNSS). Moreover, six calibration canvases (1.2 × 1.2 m^2^) with reflectance intensities at 3%, 6%, 12%, 22%, 48% and 64% were placed within the study area for radiometric calibration. The flights were conducted at the altitude of 50 m above ground with a flight speed of 3 m/s to obtain images at the spatial resolution of 3 cm during the three critical growth stages including jointing (27 March), heading (12 April), anthesis (22 April) in 2017. The range of solar zenith angles and solar azimuth angles were 50°–60° and 130°–230° for jointing stage, 54°–66° and 124°–236° for heading stage, 56°–70° and 130°–240° for anthesis stage, respectively. All flights were carried out in stable ambient light conditions between 10:00 and 14:00 local time with the fixed flight speed and route planning during the entire season.

#### Determination of N Nutrition Parameters

Destructive sampling of the 36 plots was conducted within one day of each UAV campaign. Thirty plants were randomly harvested from each sampling region to represent the homogenous plot and then separated into leaves, stems and panicles (panicles for heading and anthesis stages only). All the samples were oven-dried at 105°C for 30 min and afterwards at 80°C for about 48 h until a constant weight. The dried wheat organs (leaves, stems and panicles) were weighed, ground and stored in plastic bags for chemical analysis. Moreover, the number of plants per unit ground area was manually counted to extrapolate the leaf, stem and panicle biomass of the entire plot by following the procedures in Lu et al. (2019). The N concentration in the leaf, stem and panicle tissues was separately determined from 0.15 g of dried samples with the micro-Keldjahl method (Bremner and Mulvaney, 1982). The N concentration in the leaf, stem and panicle tissues was determined with the micro-Keldjahl method (Bremner and Mulvaney, 1982). The LNC (%) represented the mass of N in the leaf per unit dry weight (Li et al., 2018). Above-ground biomass (AGB) was calculated as the sum of dry biomass in leaves, stems and panicles per unit ground area. The N accumulation (g/m^2^) of leaves (LNA) and plants (PNA) was calculated as the product of N concentration (%) and leaf dry biomass (t/ha) and AGB, respectively (Zheng et al., 2018a). The PNC (%) was derived from the ratio of PNA to AGB. These variables were determined with the equations below:

(1) $$AGB=B_{leaf}+B_{stem}+B_{panicle}$$

(2) $$LNA=LNC\times B_{leaf}$$

(3) $$PNA=LNC\times B_{leaf}+N_{stem}\times B_{stem}+N_{panicle}\times B_{panicle}$$

(4) PNC=PNA/AGB

where N_stem_ and N_panicle_ represents the N concentration of the stem and panicle per unit dry weight, respectively. B_leaf_, B_stem_ and B_panicle_ represent dry biomass of the leaves, stems and panicles per unit ground area. The basic statistics of N nutrition characteristics are shown in **Table 1**.

**Table 1:** Basic statistics of the LNC, PNC, LNA and PNA of wheat.

Stage	LNC (%)				PNC (%)				LNA (g/m^2^)				PNA (g/m^2^)			
	Min	Mean	Max	Std	Min	Mean	Max	Std	Min	Mean	Max	Std	Min	Mean	Max	Std
Jointing	1.84	2.48	3.04	0.32	1.21	1.58	2.19	0.22	0.81	2.43	7.74	1.53	1.54	4.04	11.51	2.36
Heading	1.92	3.30	4.16	0.62	0.97	1.76	2.80	0.46	1.14	3.56	9.95	2.09	2.43	8.04	21.07	4.42
Anthesis	2.01	3.22	4.48	0.66	0.80	1.38	1.96	0.34	1.10	3.98	9.37	2.17	2.94	9.92	21.86	4.97
All	1.84	3.0	4.48	0.67	0.80	1.57	2.80	0.39	0.81	3.32	9.95	2.06	1.54	7.33	21.86	4.77

### Image Pre-Processing and Spectral Vegetation Index Calculation

**Figure 3:**
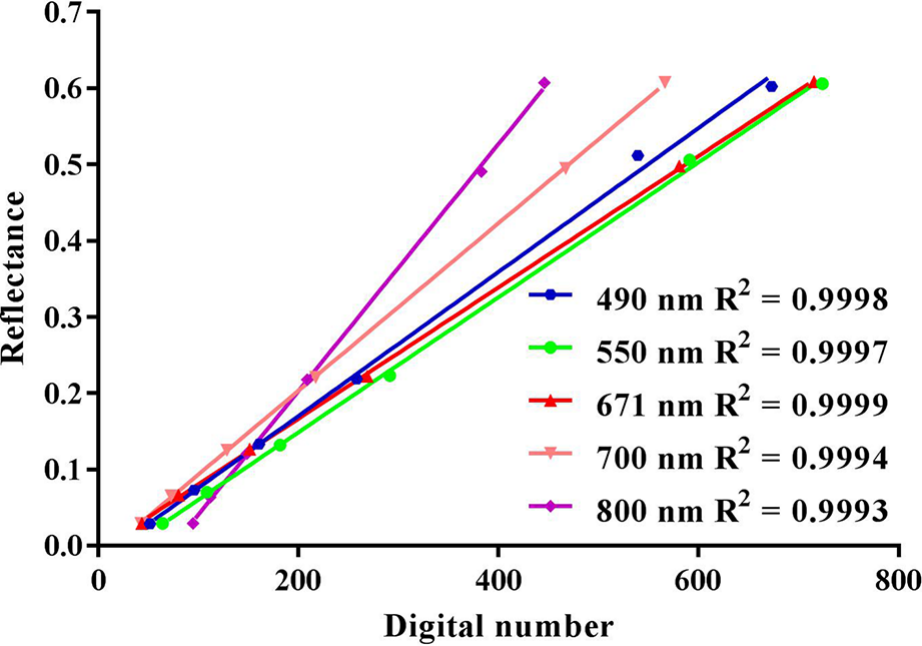
Relationships between digital number and the reflectance of calibration panels for the five bands of the UAV-based multispectral camera.

The pre-processing workflow of UAV images included noise reduction, vignetting correction, lens distortion correction (Kelcey and Lucieer, 2012), band by band alignment (Turner et al., 2014) and radiometric calibration (Smith and Milton, 1999). To reduce the band-to-band misalignment, we manually registered the five bands with 25 GCPs marked on the concrete roads. The registered images were stacked into one five-band image in TIFF format. In addition, the digital number (DN) values of the images were transformed into reflectance values per band by applying the empirical line model derived from the measured reflectance values and DN values of the six calibration canvases (**Figure 3**). A region of interest (ROI) of the fixed size was delineated in the non-sampling area of each plot, which was applied for each flight campaign. The mean value of each ROI extracted from the reflectance image for each growth stage was used to represent the reflectance of each plot. All pixels in each ROI were used regardless of vegetation or non-vegetation pixels. Therefore, we had 36 samples for each stage and a total of 108 samples for the three growth stages for subsequent analysis. The image pre-processing and radiometric calibration were mostly performed in the IDL/ENVI environment (Exelis Visual Information Solutions, Boulder, CO, USA), ArcGIS 10.2.2 (Esri, Redlands, CA, USA). We examined four published VIs derived from the five-band images for the estimation of N nutrition status (**Table 2**). The visible atmospherically resistant index (VARI) derived from the visible region has proved to be sensitive to vegetation fraction (Gitelson et al., 2002) and correlate well with LAI and biomass (Gitelson et al., 2003b). The modified normalized difference index with a blue band (mND_blue_) was proposed by Jay et al. (2019) as a strong indicator of crop chlorophyll content with weak effect of soil background. The green band chlorophyll index (CI_green_) and the red edge chlorophyll index (CI_red-edge_) have proved to be accurate predictors of leaf (Gitelson et al., 2003a; Schlemmer et al., 2013) and canopy chlorophyll contents (Gitelson 2005; Schlemmer et al., 2013; Clevers et al., 2017). These VIs were selected to represent the differences in band combination, and sensitivity to soil background, biomass and chlorophyll content (Zheng et al., 2018a).

**Table 2:** Vegetation indices used in this study.

Full name	Abbreviation	Formulation	Reference
Visible Atmospherically Resistant Index	VARI	(R_550_-R_671_)/(R_550_+R_671_-R_490_)	Gitelson et al. (2002)
Red edge Chlorophyll Index	CI_red-edge_	R_800_/R_700_-1	Gitelson et al. (2003b)
Green band Chlorophyll Index	CI_green_	R_800_/R_550_-1	Gitelson et al. (2003b)
Modified Normalized Difference Vegetation Index	mND_blue_	(R_490_-R_700_)/(R_490_+R_800_)	Jay et al. (2017a)

The formulation of VIs was derived from references, but the use of waveband was based on our multi-spectral camera.

### Model Calibration and Validation

To form a large and comprehensive dataset, the samples collected for all plots from the three critical growth stages were pooled together. The pooled dataset was split into two parts, with 70% for model calibration and the remainder 30% for model validation. A linear regression model was used to evaluate the performance of the VIs. To evaluate the combination of nadir-view and oblique-view images, a two-variable regression was employed to establish multi-angular models. The calibrated models were evaluated with the accuracy metrics coefficient of determination (*R^2^*) and the root mean square error (RMSE) determined for the validation data. All these procedures were implemented in R x64 3.4.0 environment (R Development Core Team, 2017).

## Results

### Variation in Canopy Reflectance of Wheat With VZA

**Figure 4:**
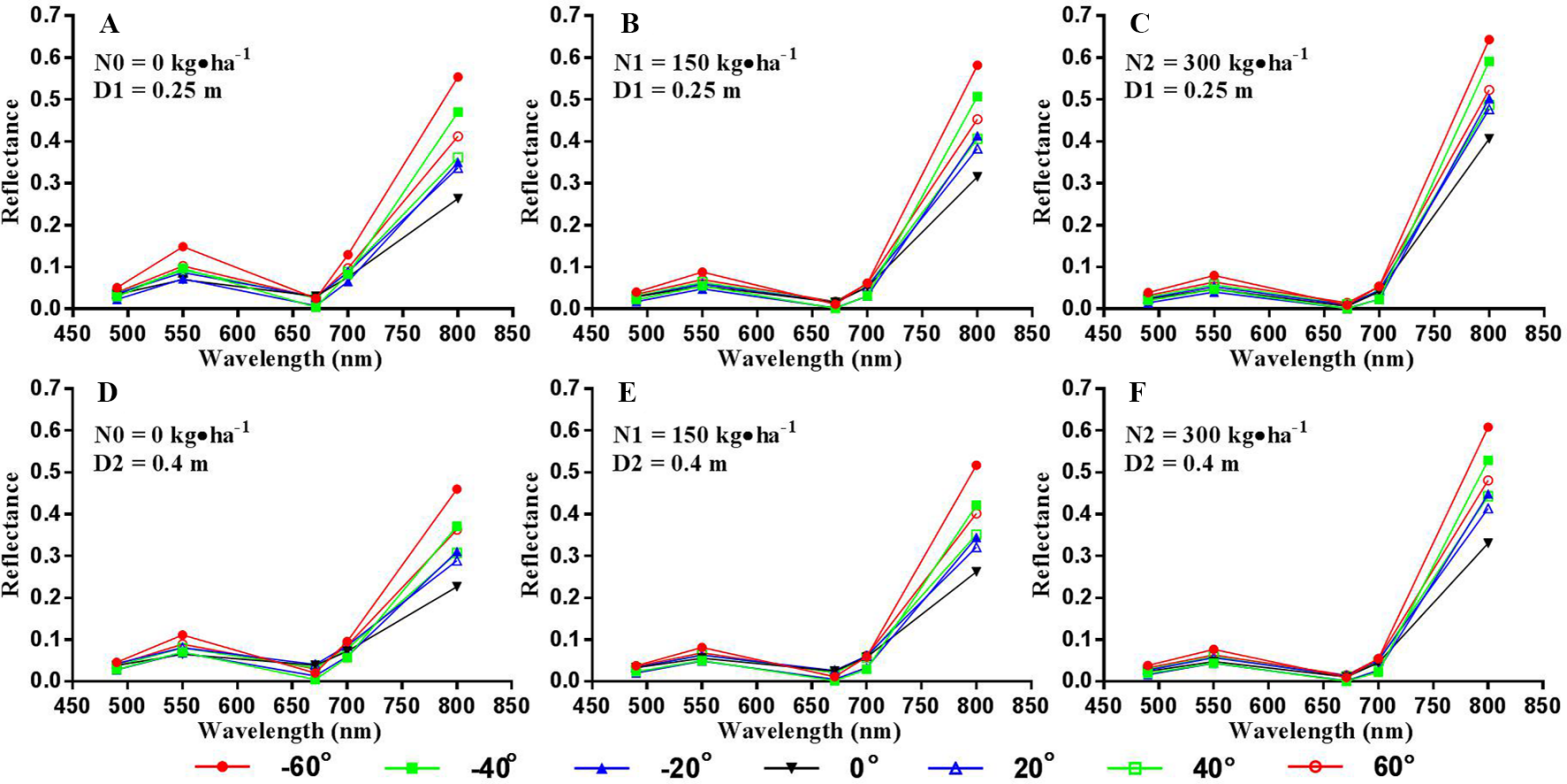
Canopy spectral reflectance of winter wheat over different viewing angles for the erectophile variety with different treatments (A: D1N0, B: D1N1, C: D1N2, D: D2N0, E: D2N1, F: D2N2; D1 = 0.25 m, D2 = 0.4 m; N0 = 0 kg•ha^-1^, N1 = 150 kg•ha^-1^, N2 = 300 kg•ha^-1^; D and N represent row spacing and nitrogen level, respectively) at the heading stage from the experimental site.

The canopy reflectance varies with VZA for every band, and the spectral variation between view angles for the NIR band (800 nm) is more significant than that for other bands (**Figure 4**). The highest reflectance was consistently observed from the backward direction of -60° among all seven viewing angles for different cultivars and N treatments. In addition, the lowest reflectance derived from the NIR band occurred at nadir observations.

**Figure 5:**
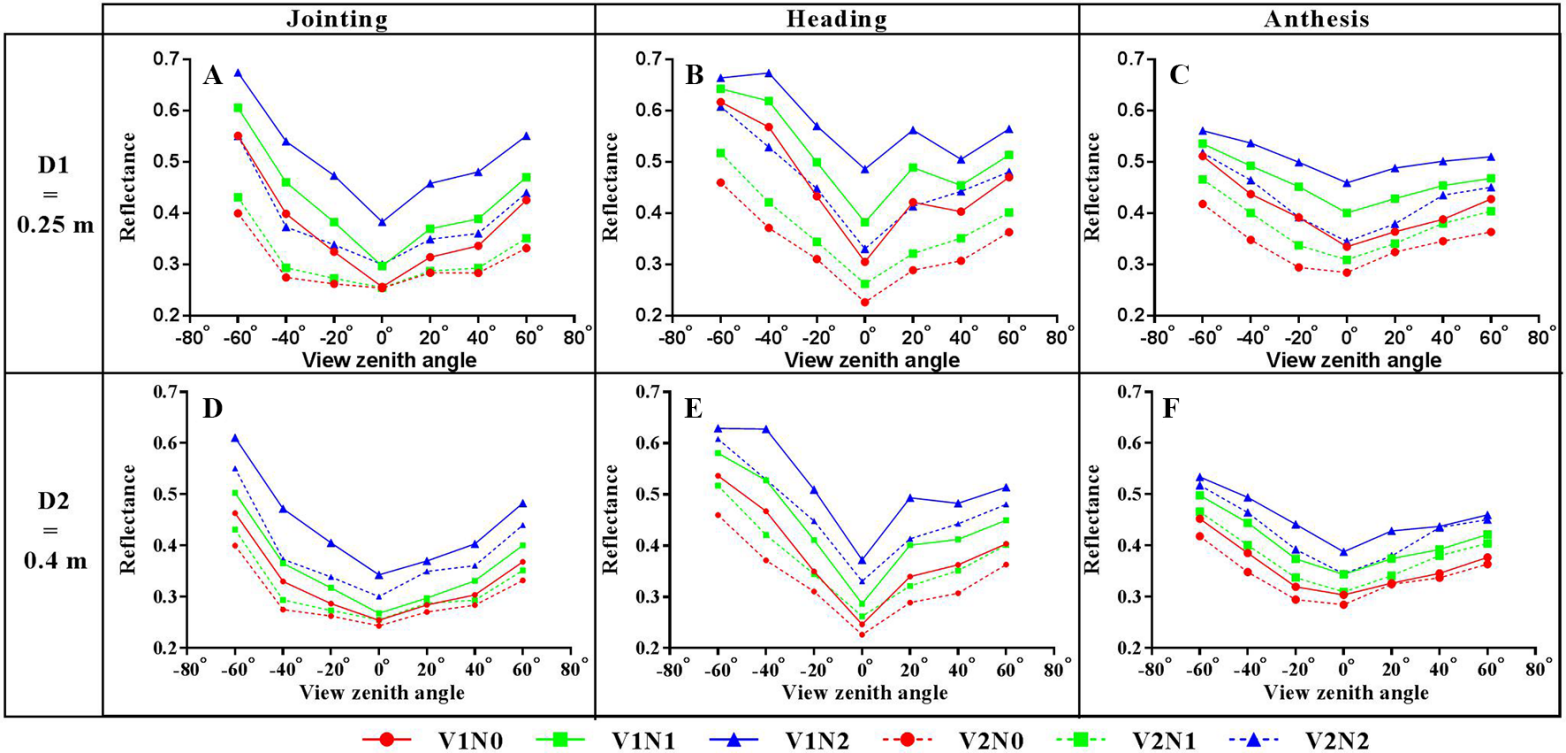
Variation of the spectral reflectance at 800 nm with VZA for different growing stages of winter wheat (left column: Jointing **(A**, **D)**, middle column: Heading **(B**, **E)**, right column: Anthesis **(C**, **F)**; top row: D1 = 0.25 m, bottom row: D2 = 0.4 m; V1 = planophile type, V2 = erectophile type; N0 = 0 kg•ha^-1^, N1 = 150 kg•ha^-1^, N2 = 300 kg•ha^-1^; V, D, N represent variety, row spacing and nitrogen level, respectively).

**Figure 5** shows the variation of spectral reflectance at the NIR band with VZA for different treatments and growth stages. The trend of canopy reflectance with VZA exhibits the bowl effect (Wang et al., 2012; Song et al., 2016), with the reflectance at 0° as the bowl bottom and decreasing bowl depth from jointing to anthesis stages. In addition, the reflectance derived from the viewing angles in the backward direction was generally higher than their counterparts in the forward direction. The planophile variety (V1) exhibited consistently higher canopy reflectance than the erectophile variety (V2) with the same nitrogen, density and growth stage.

### Performance of VIs for LNC and PNC Estimation at Different VZAs

**Figure 6:**
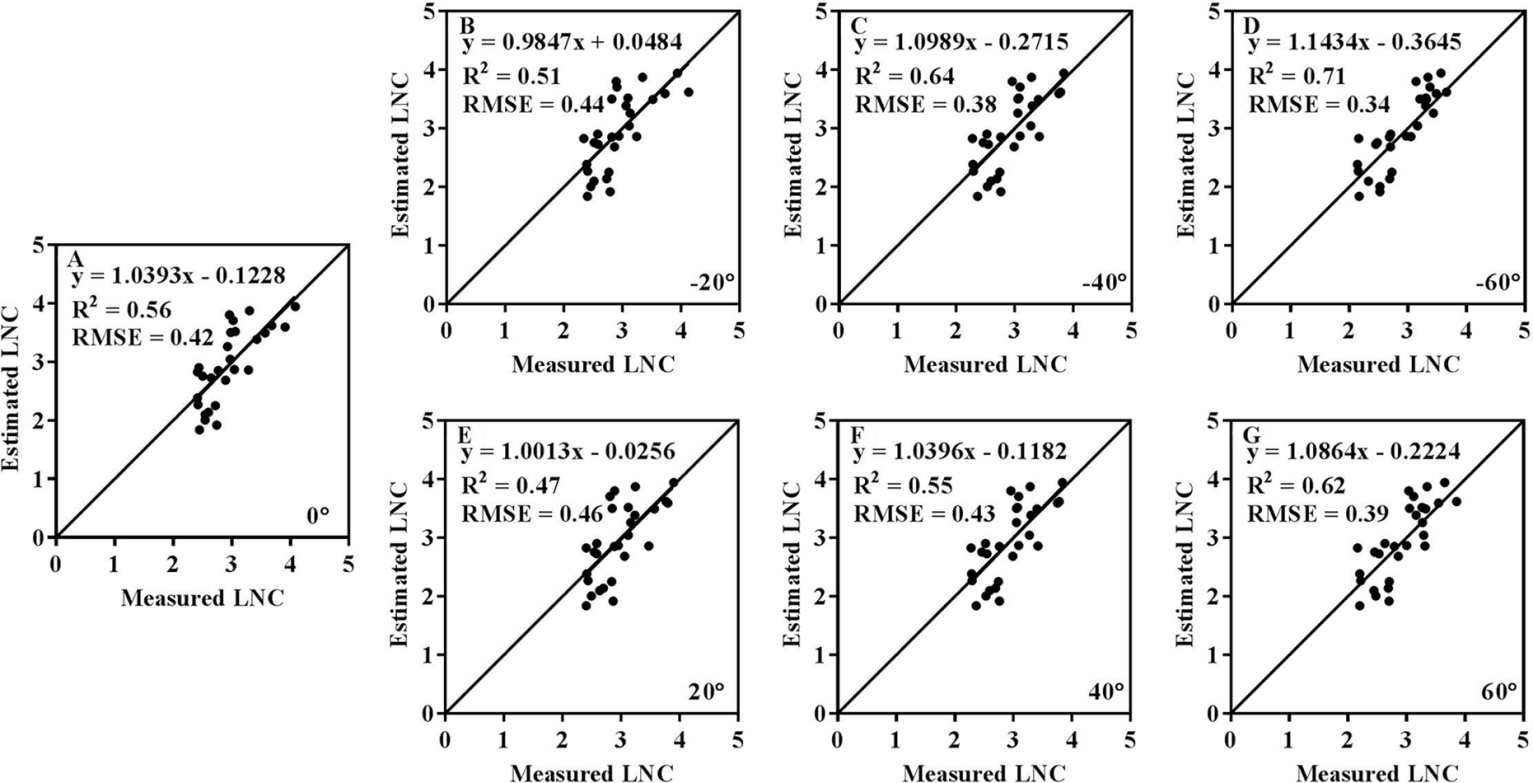
Comparison between measured and estimated LNC values with CIgreen at seven VZAs (A: 0°, B: -20°, C: -40°, D: -60°, E: 20°, F: 40°, G: 60°).

A summary of *R^2^* and RMSE values derived from the relationships between four spectral indices and LNC at different VZAs is shown in **Table 3**. Obviously, the estimation accuracy varied with VZA for each of the VIs. Among the four VIs, CI_green_ yielded the highest accuracy for most VZAs (**Figure 6**) and VARI exhibited the lowest accuracy for all VZAs. mND_blue_ and CI_red-edge_ achieved moderate accuracies, with the former being superior to the latter from -40° to 40°. Overall, the estimation accuracy was higher for backward VZAs than that for the corresponding forward VZAs. Significantly higher accuracies were yielded with CI_green_ (*R^2^* = 0.71, RMSE = 0.34%) and CI_red-edge_ (*R^2^* = 0.65, RMSE = 0.37%) at -60° than those at other VZAs.

**Table 3:** Coefficient of determination (*R^2^*) and RMSE (%) for the relationships between four spectral indices and LNC or PNC at different viewing zenith angles.

Variable	VZA	mND_blue_		CI_green_		CI_red-edge_		VARI	
		*R^2^*	RMSE	*R^2^*	RMSE	*R^2^*	RMSE	*R^2^*	RMSE
LNC	–60°	0.55	0.42	**0.71**	**0.34**	**0.65**	**0.37**	0.34	0.52
	–40°	0.60	0.40	0.64	0.38	0.44	0.47	0.37	0.50
	–20°	0.62	0.39	0.51	0.44	0.49	0.45	0.43	0.48
	0°	**0.65**	**0.37**	0.56	0.42	0.54	0.43	**0.46**	**0.47**
	20°	0.55	0.42	0.47	0.46	0.44	0.47	0.24	0.55
	40°	0.58	0.41	0.55	0.43	0.50	0.45	0.24	0.55
	60°	0.55	0.43	0.62	0.39	0.55	0.42	0.13	0.59
PNC	–60°	0.09	0.35	0.24	0.32	0.30	0.31	0.21	0.32
	–40°	0.11	0.34	**0.36**	**0.29**	**0.35**	**0.29**	0.22	0.32
	–20°	0.12	0.34	0.25	0.32	0.29	0.31	0.19	0.33
	0°	**0.28**	**0.31**	0.27	0.31	0.30	0.31	**0.32**	**0.30**
	20°	0.20	0.33	0.22	0.32	0.19	0.33	0.21	0.32
	40°	0.24	0.32	0.28	0.31	0.29	0.31	0.19	0.33
	60°	0.07	0.35	0.28	0.31	0.20	0.33	0.08	0.35

The accuracy metrics were calculated from validation data. The number in bold represents the maximum R^2^ and minimum RMSE (%), respectively.

**Figure 7:**
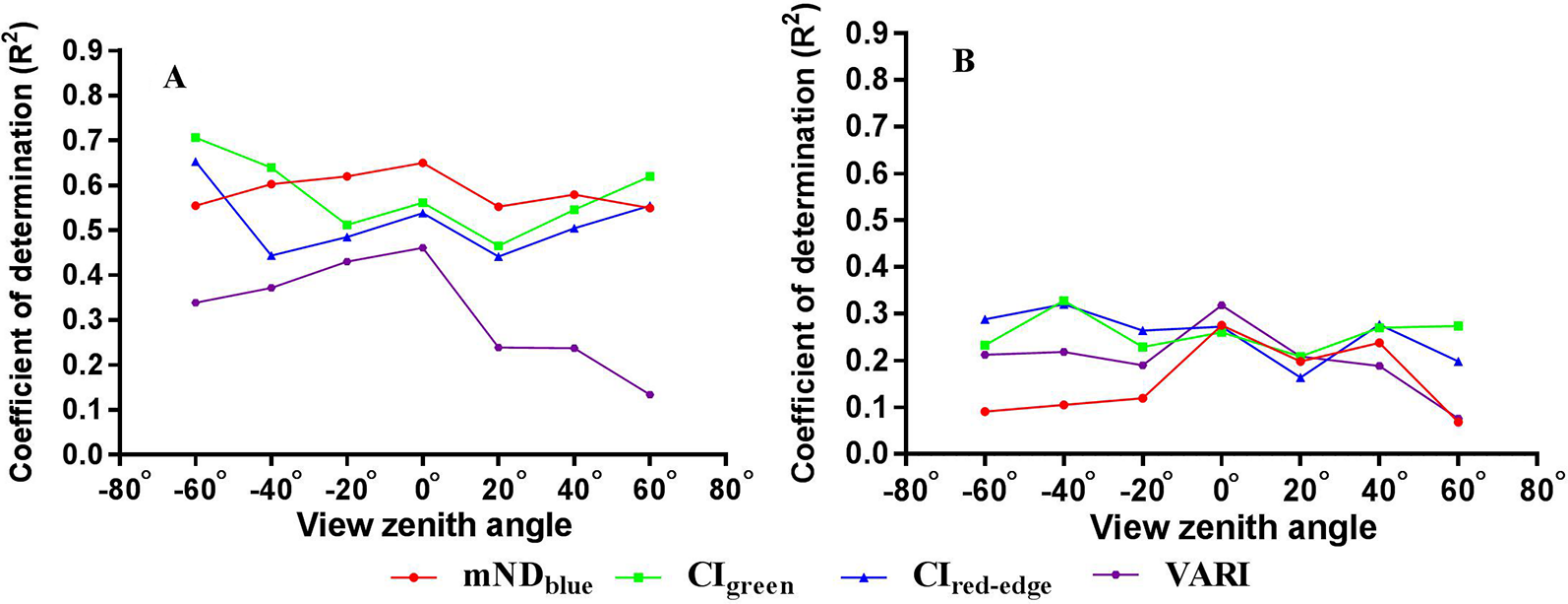
Coefficient of determination (*R^2^*) values for the relationships of four VIs with **(A)** LNC and **(B)** PNC over the seven VZAs.

The performance of four VIs with VZA for estimating LNC and PNC from four VIs is shown in **Figure 7**. For LNC estimation, the best performance was obtained at -60° for all VIs. Compared to other VIs, mND_blue_ exhibited higher *R^2^* values from -20° to 20°. The highest estimation accuracy for VARI and mND_blue_ was obtained at nadir observation and the accuracy decreased dramatically with VZA in the backward and forward directions, respectively. For CI_green_ and CI_red-edge_, the *R^2^* values decreased when the VZA varied from 0° to 20° and generally increased when the VZA varied from 20° to 60° in both directions. For PNC estimation, the best performance (*R^2^* < 0.35) was obviously lower than that for LNC performance for all VIs (*R^2^* > 0.71). The highest *R^2^* occurred at -40° for CI_green_ and CI_red-edge_ but 0° for mND_blue_ and VARI.

### Performance of VIs for LNA and PNA Estimation at Different VZAs

**Figure 8:**
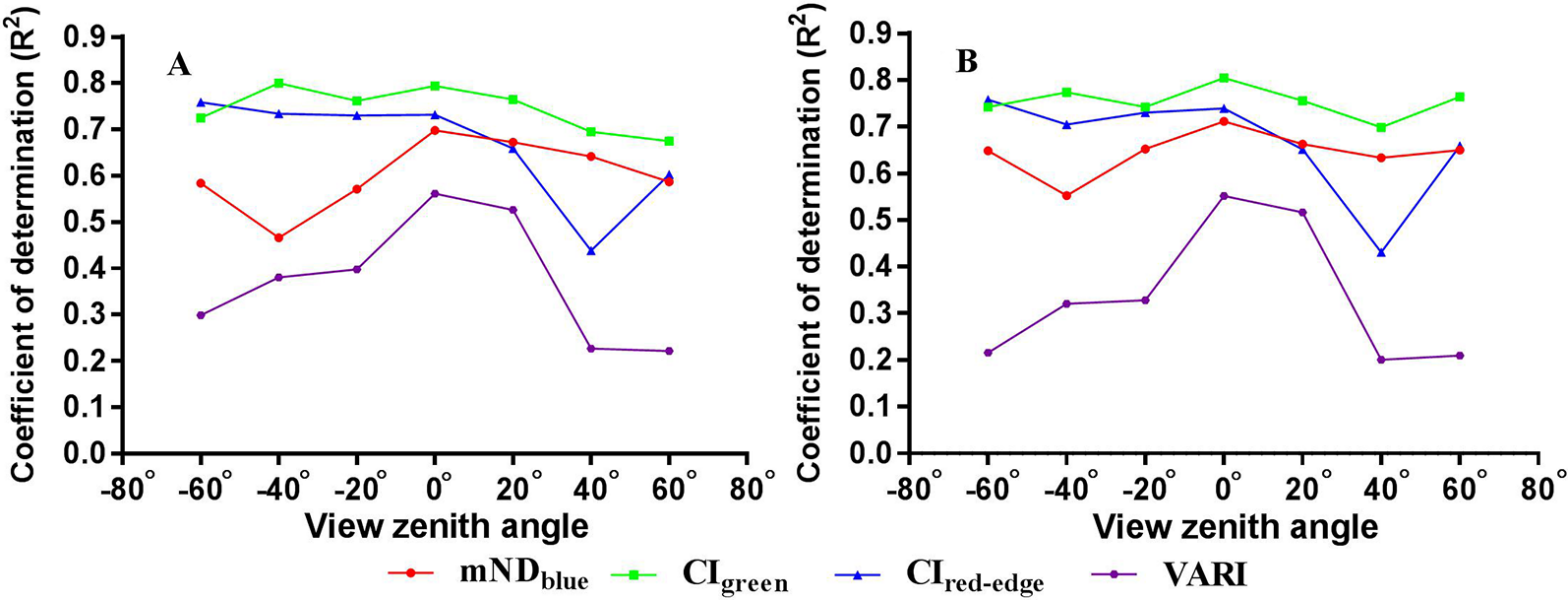
Coefficient of determination (*R^2^*) values for the relationships of four VIs with **(A)** LNA and **(B)** PNA over the seven VZAs.

The variation in the coefficient of determination (*R^2^*) with VZA for the estimation of LNA and PNA from four VIs is presented in **Figure 8**. For LNA estimation, CI_green_ and VARI exhibited the least sensitivity and greatest sensitivity to VZA, respectively. Their *R^2^* profiles with regard to VZA also exhibited the highest and lowest values. The *R^2^* profile for CI_red-edge_ was stable for the VZAs from -60° to 0° but exhibited a significant valley at 40°. In contrast, the mND_blue_ achieved the highest *R^2^* at nadir observation and also was sensitive to VZA. Similar patterns were observed for the estimation of PNA.

### Angle Combinations for LNC and PNC Estimation

**Table 4** lists the *R^2^* values derived with four VIs from a combination of 0° and an off-nadir VZA for LNC and PNC estimation, with the *R^2^* values derived from 0° as the baseline. For LNC estimation, the combination of 0° and an off-nadir VZA improved the estimation accuracy for CI_green_, CI_red-edge_ and VARI as compared to only using a nadir VZA. Among all VIs and all angle combinations, the highest accuracy was achieved with CI_green_ from the combination of 0° and -60° (*R^2^* = 0.66, RMSE = 0.39%). For PNC estimation, the combination of 0° and an off-nadir VZA obviously performed better than the nadir VZA for all VIs. Although all *R^2^* values for PNC estimation were lower than their counterparts for LNC estimation, the improvements from angle combinations for the former were generally more significant than those for the latter. The highest accuracy in the estimation of PNC was achieved with CI_red-edge_ from the combination of 0° and -40° (*R^2^* = 0.52, RMSE = 0.28%).

**Table 4:** Coefficient of determination (*R^2^*) and RMSE (%) for LNC and PNC estimation using the VIs from a combination of 0° and other off-nadir VZAs.

Variable	VZA combination	mND_blue_		CI_green_		CI_red-edge_		VARI	
		*R^2^*	RMSE	*R^2^*	RMSE	*R^2^*	RMSE	*R^2^*	RMSE
LNC	0° + (-60°)	0.54	0.45	**0.66**	0.39	**0.62**	0.41	0.27	0.57
	0° + (-40°)	0.56	0.44	0.58	0.43	0.45	0.49	0.23	0.58
	0° + (-20°)	0.54	0.45	0.48	0.48	0.48	0.48	0.23	0.58
	0° + 20°	0.55	0.44	0.47	0.50	0.41	0.51	0.23	0.58
	0° + 40°	0.54	0.45	0.48	0.48	0.41	0.51	0.27	0.57
	0° + 60°	0.56	0.44	0.55	0.44	0.55	0.44	0.20	0.59
	0°	**0.65**	**0.37**	0.56	0.52	0.54	0.43	**0.46**	0.47
PNC	0° + (-60°)	0.40	0.31	**0.47**	**0.29**	0.48	0.29	0.41	0.31
	0° + (-40°)	0.37	0.32	0.47	0.30	**0.52**	**0.28**	0.41	0.31
	0° + (-20°)	0.40	0.31	0.47	0.29	0.50	0.29	0.41	0.31
	0° + 20°	0.41	0.31	0.45	0.30	0.48	0.29	0.40	0.31
	0° + 40°	0.46	0.30	0.47	0.30	0.47	0.29	0.42	0.31
	0° + 60°	**0.49**	**0.29**	0.46	0.30	0.50	0.29	**0.43**	0.31
	0°	0.28	0.31	0.26	0.31	0.27	0.31	0.33	0.32

The number in bold represents the maximum R^2^ calculated for each nutrition parameter and VI. The row of 0° corresponds to the estimation accuracies for VIs from the nadir only.

### Angle Combinations for LNA and PNA Estimation

The corresponding *R^2^* and RMSE values for LNA and PNA estimation are presented in **Table 5**. The addition of off-nadir VZAs did not improve the performance of each VI in LNA estimation, and the accuracies obtained at the nadir VZA were higher than those in LNC estimation for all VIs. The highest accuracy for LNA was observed for CI_green_ from the nadir view (*R^2^* = 0.79, RMSE = 0.83 g/m^2^). For PNA estimation, the addition of off-nadir VZAs could slightly improve the performance of mND_blue_ and CI_red-edge_. However, the best performance was still achieved with CI_green_ from the nadir observation (*R^2^* = 0.80, RMSE = 1.80 g/m^2^).

**Table 5:** Coefficient of determination (*R^2^*) and RMSE (g/m^2^) for LNA and PNA estimation using the VIs from a combination of 0° and an off-nadir VZA.

Variable	VZA combination	mND_blue_		CI_green_		CI_red-edge_		VARI	
		*R^2^*	RMSE	*R^2^*	RMSE	*R^2^*	RMSE	*R^2^*	RMSE
LNA	0° + (-60°)	0.64	1.14	0.72	1.00	0.64	1.13	0.51	1.32
	0° + (-40°)	0.66	1.09	0.69	1.05	0.63	1.15	0.51	1.32
	0° + (-20°)	0.67	1.08	0.63	1.15	0.63	1.15	0.48	1.37
	0° + 20°	0.60	1.19	0.62	1.16	0.54	1.27	0.49	1.34
	0° + 40°	0.60	1.19	0.68	1.06	0.62	1.16	0.50	1.33
	0° + 60°	0.61	1.18	0.69	1.06	0.70	1.04	0.53	1.29
	0°	**0.70**	1.00	**0.79**	0.83	**0.73**	0.94	**0.56**	1.20
PNA	0° + (-60°)	**0.79**	2.10	0.81	2.02	0.75	2.27	0.54	3.08
	0° + (-40°)	0.78	2.15	0.78	2.13	0.76	2.26	0.50	3.23
	0° + (-20°)	0.78	2.12	0.78	2.12	0.78	2.15	0.51	3.19
	0° + 20°	0.75	2.29	0.77	2.18	0.77	2.21	0.58	2.97
	0° + 40°	0.76	2.25	0.77	2.19	0.74	2.33	0.59	2.94
	0° + 60°	0.74	2.32	0.80	2.05	**0.80**	2.05	0.56	3.02
	0°	0.71	2.18	**0.80**	1.80	0.74	2.07	**0.55**	2.72

The number in bold represents the maximum R^2^ calculated from validation data for each agronomic variable with VI. The row of 0° corresponds to the estimation accuracies for VIs from the nadir VZA only.

## Discussion

### The Effect of VZA on N Status Estimation

The performance of VIs in the estimation of N nutrition status was dependent not only on the selected bands, but also the VZA (**Figure 5**). Overall, the VIs derived from the backward direction performed better than those from the forward direction. Huber et al. (2007) also demonstrated that mono-directional models derived from backward viewing angles performed better than those from forward viewing angles for the prediction of foliar nitrogen concentration. This could be because stronger spectral signals of N dynamics were obtained from the sunlit stems and leaves in the backward direction than those from shady stems and leaves in the forward direction (Verrelst et al., 2008; He et al., 2016b). Since the highest reflectance could be obtained at the hotspot position from the backward viewing direction where the sensor is aligned with the sun (Jupp and Strahler, 1991), some studies attempted to improve the estimation of vegetation parameters using the hotspot effect (Pocewicz et al., 2007; Meng et al., 2016). However, it is difficult to accurately capture the hotspot reflectance because it depends not only on crop canopy architecture, but also on the VZA (Chen et al., 2003; Liao et al., 2013). Given the labor and time cost in capturing the hotspot effect, we adopted the 20° angle sampling interval that was unable to present the hot spot reflectance while analyzing the directional effect. In addition, the magnitude of the canopy hotspot effect largely depends on the size, shape, orientation, density and spatial dispersion of leaves (Qin and Xiang, 1994). Although the reflectance at hotspot direction is valuable for estimating canopy structural parameters, such as clumping index (Chen et al., 2005), this study focused on the estimation of canopy chemistry for practical application purposes by examining the optimal single or dual observation angles.

Moreover, the VZA affected the possibility of sensing the soil background from the UAV platform especially for early growth stages. The NIR band exhibited stronger spectral difference between VZAs than other bands (i.e. 490 nm, 550 nm, 671 nm, and 700 nm) (**Figure 4**), which was caused by the spatial heterogeneity of spectral properties within the field of view. Generally, a higher VZA led to more chances to sense the vegetative parts and fewer chances to sense the soil background in the winter wheat fields. As shown in **Figure 4**, the highest canopy reflectance at the NIR band was obtained from the -60° image, while the lowest value was at nadir. Our finding is consistent with the previous study by Verrelst et al. (2008) who demonstrated that the VIs were significantly affected by the proportions of soil background and vegetation within the field of views. Compared to the off-nadir ones, the nadir observations had more signals from the soil background due to the existence of canopy gaps. The off-nadir observations corresponded to different proportions of soil background seen from the sensor, which could explain the difference in canopy reflectance between VZAs.

### Difference in Estimation Accuracy Between VIs

Among the four VIs examined, VARI performed worst for the estimation of N nutrition parameters over all VZAs and CI_green_ performed best. Since VARI was constructed from visible bands alone, the lack of the NIR band enhancing the contrast between different N levels might be responsible for the worst performance (Hunt et al., 2010). The superior performance of CI_green_ over CI_red-edge_ and mND_blue_ was probably attributed to the greater sensitivity of the green band to chlorophyll concentration than the red edge (RE) band. In addition, the wavelength center of the RE band (700 nm) as compared to 720~730 nm used in other studies was only approximately 30 nm offset the red band (671 nm) (Feng et al., 2015; Zhou et al., 2018). Considering the 10 nm bandwidth of these bands in the multispectral camera, the potential of the RE band may need to be further explored with longer wavelengths.

For the estimation of LNA and PNA, the accuracy for the VARI at nadir position was remarkably better than those at off-nadir positions (except for VZA = 20°). The mND_blue_ exhibited stronger sensitivity to VZA in backward direction than forward direction. Due to the influence of the dark green pixels from shaded leaves, the mND_blue_ yielded higher *R^2^* in the forward viewing direction compared to the backward direction. This was similar to the finding reported by Jay et al. (2019) on quantifying the biochemistry in sugar beet crops. In contrast, the other two VIs (CI_red-edge_, CI_green)_ appeared to be much less sensitive to VZA. This was especially the case for the two VIs at backward viewing directions (**Figure 8**). Specifically, VARI was composed of merely visible bands and the other three included the NIR band. The high-absorbing visible bands in the VARI at off-nadir positions might saturate for the estimation of biomass. The main reason for the insensitivity of the CI_red-edge_ and CI_green_ to VZA was probably that leaf or plant biomass contributed dominantly to the N accumulation variables and the performance of biomass sensitive VIs were insignificantly dependent on VZA (Gianelle and Guastella, 2007). The biomass signals of crop canopies were strong for the growth stages examined in this study and did not need to be enhanced from off-nadir observations. Overall, the estimation accuracies for N accumulation (LNA and PNA) were obviously higher than those for N concentration (LNC and PNC). The canopy-level finding was supported by a recent study by Li et al. (2018) who demonstrated that it was easier to estimate N mass per unit surface area than that per unit dry mass. This was explained by the better representation of the interaction between matter and light per unit surface area with spectral reflectance from leaf optical properties perspective.

Given the links between agronomic variables, one may be concerned about the dominant role of biomass in the estimation of N status. To address this issue, we analyzed their relationships and found the nitrogen accumulation was strongly correlated to biomass, both for leaves (LNA vs leaf biomass: *R^2^* = 0.85, p < 0.0001) and the plants (PNA vs above-ground biomass: *R^2^* = 0.79, p < 0.0001). However, the nitrogen concentration was only weakly correlated to biomass for leaves (LNC vs leaf biomass: *R^2^* = 0.23, p < 0.0001) and not correlated to biomass for plants (PNA vs above-ground biomass: *R^2^* = 0.01, p > 0.42).Therefore, biomass (leaf biomass or above-ground biomass) might play a significant role in nitrogen accumulation (LNA or PNA) but not so in nitrogen concentration (LNC or PNC). Although the variation of accuracy across VZAs for biomass estimation (**Table 6**) was almost consistent with that for LNA and PNA, the accuracies for the former were lower than those for the latter under the same VI and SZA. Biomass might have played a dominant role in nitrogen accumulation, but definitely not in nitrogen concentration. In addition, the accuracies for nitrogen accumulation were already high with nadir observations alone. The improvements for LNC and PNC estimation by using oblique observations should be attributed to the stronger capability of detecting nitrogen in the leaves or the canopies.

**Table 6:** Coefficient of determination (*R^2^*) and RMSE (t/ha) for AGB and leaf biomass estimation using the VIs from seven VZAs.

Variable	VZA	mND_blue_		CI_green_		CI_red-edge_		VARI	
		*R^2^*	RMSE	*R^2^*	RMSE	*R^2^*	RMSE	*R^2^*	RMSE
AGB	–60°	0.60	1.52	0.50	1.70	0.57	1.58	0.18	2.18
	–40°	0.51	1.69	0.51	1.68	0.44	1.80	0.18	2.18
	–20°	0.54	1.63	0.50	1.71	0.49	1.72	0.17	2.19
	0°	0.48	1.74	0.53	1.66	0.45	1.78	0.29	2.03
	20°	0.53	1.65	0.49	1.72	0.37	1.90	0.36	1.92
	40°	0.44	1.80	0.43	1.82	0.20	2.16	0.09	2.30
	60°	0.60	1.52	0.56	1.60	0.43	1.83	0.10	2.29
Leaf biomass	–60°	0.54	0.30	0.62	0.28	0.68	0.25	0.38	0.35
	–40°	0.54	0.30	0.72	0.24	0.66	0.26	0.41	0.34
	–20°	0.58	0.29	0.69	0.25	0.65	0.26	0.41	0.34
	0°	0.58	0.29	0.67	0.26	0.60	0.28	0.47	0.32
	20°	0.50	0.31	0.65	0.26	0.54	0.30	0.47	0.32
	40°	0.40	0.34	0.56	0.29	0.34	0.36	0.17	0.40
	60°	0.48	0.32	0.53	0.31	0.47	0.32	0.19	0.40

### The Angular Combinations for the Estimation of Agricultural Parameters

Given the vertical variation in N concentration within the crop canopy, the N concentration derived from the top canopy is obviously different than that from the whole plant or other positions within the canopy. The off-nadir observations have been proved to be useful in obtaining more detailed information about crop N status, especially for open canopies (Pocewicz et al., 2007; Huang et al., 2011). Our study demonstrated that the off-nadir observations could improve the estimation accuracy for LNC and PNC, which could be explained by fact that VIs from the 0° image mainly captured the information of N concentration from the upper leaves or panicles but lacked the information from the lower organs.

To investigate the contribution of multi-angular spectral data, we evaluated the performance of the combination of nadir and off-nadir images for the estimation of N nutrition parameters. For LNC estimation, a slight improvement was achieved when using the combination of 0° and -60° images as compared to the 0° image alone. Although the off-nadir observations could acquire more information within the canopy, most leaves could be sensed from the 0° VZA and no significant improvement could be obtained by adding an off-nadir image. Conversely, the combination of 0° and another angle substantially improved the estimation accuracy for PNC with each VI. Off-nadir observations created more chances to sense not just leaves and panicles at different heights, but also stems within the canopy. Since the off-nadir observations did not exhibit better performance than the nadir observation for LNA and PNA estimations, the addition of an off-nadir image to the nadir image failed to make an improvement.

Although some studies attempted to combine the spectral information from three or four VZAs for improving the estimation accuracy, the noise effect resulting from adding multiple VZAs may reduce the predictive power for the multi-angular model (Huber et al., 2007; He et al., 2016b). We might miss the combination of three or even more angles for improved estimation, but such a combination of multi-angular views will increase the burden of flight campaigns. Two angles would be a trade-off between multi-angular observations and data acquisition workload. Therefore, we only tested the combination of 0° and an off-nadir angle from the perspective of operability and efficiency.

### The Limitations and Potential Applications

Our study demonstrated that the estimation accuracies for N status parameters from backward VZAs were obviously higher than those from forward VZAs with most of VIs. This finding is consistent with previous studies using the spectral data from the backward viewing direction to improve the estimation of vegetation parameters, such as LAI in forest (Pocewicz et al., 2007), yield in soybean (Galvão et al., 2009), and LNC in wheat (He et al., 2016b). However, it is still difficult to acquire images at the exactly specified VZA in the field using a UAV system. Although a gimbal was used to guarantee the attitude of the sensor, the random wind might change the initial VZA during a UAV flight. Therefore, the performance of multi-angular VIs might be affected by the stability and the accuracy of the VZA with the UAV-based camera.

To avoid complicating the image collection process and aim for practical collection strategies, we have not investigated the effects of SZA and SAA on wheat canopy reflectance in this study. To reduce such effects, we tried our best to collect the aerial images during 10:00-14:00 local time so as to minimize the ranges of SZA (50°–70°) and SAA (130°–230°). Considering the symmetry of solar positions in the morning and afternoon along north-south orientation of wheat rows, the equivalent SAA ranged from 130° to 180° (or from 180° to 230°). These angle ranges were narrower than that of VZA that varied from -60° to 60°. Therefore, the results of this study should be less affected by the variations in SZA and SAA. Anyway, in the future, SZA and SAA effects should be further minimized by shortening measurement periods within a day or finishing the angular measurements over several days at a fixed time.

The optimal observation angles (-40° and -60°) found in this study were in the backward viewing direction and close to the angle of the hotspot position, which suggests the potential of hotspot effect in better estimating wheat N status. This match was derived from only one experiment in this study and remains to be confirmed with more experimental data or model simulations in future work. The superior performance of backward viewing angles over forward viewing angles could be explained by the stronger spectral signals of N dynamics from the sunlit leaves and panicles in the backward direction (Verrelst et al., 2008; He et al., 2016b). If the SZA is close to nadir position, more soil background would be sunlit and oblique observations are more vital to reduce soil background effect. Future work may include model simulations to examine whether -40° or -60° would be preferred angles, but an angle in the backward direction may be necessary for the goals achieved in this study.

Although soil background might affect the estimation of foliar chemistry (e.g., N status) from canopy reflectance spectra, this study did not separate soil pixels from vegetation pixels for two reasons. One was the weak visibility of soil background from off-nadir observations compared to nadir observations. While the soil background might be visible on nadir-view images that would become increasingly invisible on oblique-view images with larger VZAs in the background or forward viewing directions (**Figure 2B**). This was also one of the main motivations to perform this multi-angular UAV remote sensing study. The direct use of oblique-view images for N status estimation could avoid another special step of removing soil background, of which the quality may be subjective to the analyst. Nevertheless, the advantages of using multi-angular observations include not only less visibility of soil background, but also better sensing of non-leaf organs (e.g., panicles). On the other hand, soil background was not a strong factor for this study because the three sampling growth stages were in the middle period of the season and characterized by dense canopy cover. Actually, we tried applying a NDVI threshold (NDVI < 0.4 for soil) to separate soil pixels from vegetation pixels. Subsequently, we found the plot-level VIs before removing soil background (VIs for all pixels) were tightly correlated to those after removing soil background (VIs for green pixels), with *R^2^* values ranging 0.9956 to 0.9982 for CI_green_ and CI_red-edge_. To avoid redundancy, we did not add the separation of soil background to this study but focused on exploring the benefits of using oblique observations from the flexible UAV platform.

Lastly, the multispectral camera included only five bands and we could construct a limited number of sensitive VIs for N status monitoring. This might explain why our best accuracy for LNC estimation was lower than that with a hyperspectral sensor (He et al., 2016a). Although our models developed with index-based statistical approaches are dataset-specific and might lack transferability to other sites with different vegetation types, our study demonstrated the importance of considering UAV-based multi-angular observation in N monitoring. Thus, the future research may focus on the investigation of a UAV-based multi-angular hyperspectral camera for more accurate estimation of crop N status parameters.

## Conclusions

This study evaluated the performance of four representative VIs derived from the UAV-based multi-angular multispectral images for the estimation of N nutrition parameters in winter wheat. We found that the general superiority of backward viewing VIs over the forward viewing VIs was obvious for LNC, but not so for PNC. The highest accuracy was obtained with CI_green_ for LNC from a VZA of -60° (*R^2^* = 0.71, RMSE = 0.34%) and PNC from a VZA of -40° (*R^2^* = 0.36, RMSE = 0.29%). CI_green_ also yielded the highest accuracy for the estimation of LNA and PNA, but did not exhibit significant sensitivity to VZA. For mono-angular observations, the N status in concentration was more poorly estimated as compared to that in accumulation. By combining an off-nadir image and the 0° image, we were able to increase the accuracy of PNC substantially with CI_red-edge_ (*R^2^* = 0.52, RMSE = 0.28%). Although the highest accuracy for LNC estimation with dual-angle images was achieved with CI_green_ from the combination of 0° and -60° (*R^2^* = 0.66), this was still not better than that using a single-angle image (-60°) with the same index (*R^2^* = 0.71). Compared to the use of single-angle images, the improvement from the use of dual-angle images was not so significant for LNC and especially for LNA and PNA.

The findings of this study suggest that it is useful for UAV users to acquire multispectral images at oblique angles, especially in the backward viewing direction, for more accurate estimation of N concentration parameters in winter wheat. The oblique-view images could be used alone or combined with the commonly used nadir-view images for improved estimation of winter wheat N concentration. Although this study demonstrates the great potential of multi-angular multispectral imagery in UAV-based winter wheat N status monitoring, the performance of oblique observations should be further validated with other multispectral cameras popular in the community and applications to other crops.

## Data Availability Statement

The datasets for this manuscript are not publicly available because our team is working on other publications with them and they are not ready yet for sharing. Requests to access the datasets should be directed to Tao Cheng at tcheng@njau.edu.cn.

## Author Contributions

All authors have made significant contributions to this research. TC, XY, YT, YZ and WC conceived and designed the experiments. NL, DL, WW and QZ performed the experiments, processed and analyzed the data. NL and TC wrote the manuscript. FB and SL discussed the results and revised the manuscript.

## Funding

This work was supported by grants from the National Key R & D Program of China (2016YFD0300601), National Science Foundation of China (31470084, 31671582, 31725020), the earmarked fund for Jiangsu Agricultural Industry Technology System (JATS[2018]290), the 111 project (B16026), Natural Science Fund of Jiangsu Province (SBK2019044119) and the Priority Academic Program Development of Jiangsu Higher Education Institutions (PAPD).

## Conflict of Interest

The authors declare that the research was conducted in the absence of any commercial or financial relationships that could be construed as a potential conflict of interest.
